# Increased levels of serum pigment epithelium-derived factor aggravate proteinuria via induction of podocyte actin rearrangement

**DOI:** 10.1007/s11255-018-2026-3

**Published:** 2018-12-07

**Authors:** Na Huang, Xuan Zhang, Youzhao Jiang, Hao Mei, Ling Zhang, Qiong Zhang, Jiongyu Hu, Bing Chen

**Affiliations:** 1 Department of Endocrinology, Southwest Hospital, Third Military Medical University (Army Medical University), 29 Gaotanyan Street, Chongqing, 400038 China; 2 Department of Neurosurgery, Southwest Hospital, Third Military Medical University (Army Medical University), 29 Gaotanyan Street, Chongqing, 400038 China; 3 Department of Neurosurgery, The 150th Central Hospital of PLA, 1 Huaxia Street, Luoyang, Henan 471031 China; 4 Department of Endocrinology, Banan People’s Hospital of Chongqing, 2 Xinnong Street, Chongqing, 401320 China; 5 Center for Outcomes Research and Evaluation, Yale-New Haven Hospital, New Haven, CT 06510 USA; 6 Outpatient Department, Southwest Hospital, Third Military Medical University (Army Medical University), 29 Gaotanyan Street, Chongqing, 400038 China; 7 Institute of Burn Research, State Key Laboratory of Trauma, Burns and Combined Injury, Southwest Hospital, Third Military Medical University (Army Medical University), 29 Gaotanyan Street, Chongqing, 400038 China; 8 Department of Endocrinology, State Key Laboratory of Trauma, Burns and Combined Injury, Southwest Hospital, Third Military Medical University (Army Medical University), 29 Gaotanyan Street, Chongqing, 400038 China

**Keywords:** PEDF, Actin, RhoA/ROCK1, Proteinuria, Diabetic kidney disease

## Abstract

**Purpose:**

To assess the role of serum pigment epithelium-derived factor (PEDF) in the occurrence and development of proteinuria and renal dysfunction and determine its relevant signaling pathway.

**Methods:**

We analyzed serum PEDF, creatinine, the urinary albumin-to-creatinine ratio, and renal morphology of normal or streptozotocin (STZ)-induced diabetic mice, before and after treatment with PEDF. In vitro, podocytes were stimulated with PEDF under normal or high-glucose conditions; permeability was measured by the transwell assay with fluorescein isothiocyanate (FITC)-dextran; and F-actin cytoskeleton was analyzed by phalloidin staining. Apoptosis was assessed by flow cytometry. RhoA activity and ROCK1, ZO-1, nephrin, and podocin levels were detected by Western blotting.

**Results:**

Diabetic mice exhibited a high serum PEDF level. In vivo, elevated serum PEDF led to proteinuria, increased serum creatinine, and podocyte foot process fusion in normal or diabetic mice. In vitro, both high-glucose and PEDF stimulation activated the RhoA/ROCK1 pathway in podocytes and promoted cell permeability, F-actin rearrangement, and apoptosis. Inhibition of RhoA/ROCK1 alleviated the damage from these effects.

**Conclusions:**

Elevated serum PEDF aggravates the development of proteinuria and renal dysfunction by inducing F-actin arrangement, foot process fusion, and apoptosis of podocytes in both normal and diabetic mice, and this effect may be mediated by activation of the RhoA/ROCK1 pathway.

## Introduction

Diabetic kidney disease (DKD) is a severe microvascular complication of diabetes mellitus. Recently, DKD has become the leading cause of end-stage renal disease (ESRD) followed by increasing morbidity, mortality, and healthcare burden [1]. But the mechanism of DKD has still not been fully elucidated.

In recent years, numerous studies have shown that podocyte damage plays a critical role in the pathogenesis of DKD [2–4]. Podocytes are terminally differentiated cells that form the final layer of the glomerular filtration barrier (GFB) to maintain glomerular permselectivity. The role of podocytes is highly dependent on their intricate actin-based cytoskeletal architecture [5]. Loss of these actin-driven membrane extensions is tightly connected to foot process effacement (FPE), podocyte loss, and proteinuria [6].

Pigment epithelium-derived factor (PEDF) is a 50-kDa endogenous secreted glycoprotein belonging to the serine protease inhibitor (serpin) superfamily and is closely related to angiogenesis, oxidation, inflammation, tumorigenicity, neuroprotection, and permeability activities [7, 8]. Previous studies have observed that serum PEDF levels are significantly elevated in diabetic patients [9–11]. Chen et al. demonstrated that irbesartan treatment, which reduced proteinuria in DKD patients, was accompanied by a decrease in kidney and urinary PEDF [12]. Qi et al. reported that elevated serum PEDF delayed healing of diabetic foot ulcers, and anti-PEDF antibody accelerated it in db/db mice [13]. However, Terawaki et al. found the serum PEDF concentration in ESRD patients was negatively correlated with mortality [14]. These studies seem to indicate that PEDF makes a complex contribution in the pathogenesis of diabetic complications.

There is also evidence that PEDF can regulate F-actin dynamics and increase endothelial permeability by combining with adipose triglyceride lipase in sepsis [15]. Based on the cytoskeleton-related filtration function of podocytes and the permeability-related activity of PEDF, it is reasonable to hypothesize that PEDF may be involved with the development of proteinuria.

In the current study, we aimed to elucidate the effect of PEDF on proteinuria by investigating the role of PEDF in regulating the F-actin arrangement of podocytes, and exploring the underlying molecular mechanisms. This work may help improve our understanding of the mechanisms of DKD, as well as provide new strategies for prevention and treatment.

## Materials and methods

### Antibodies

Anti-ZO-1 (zonula occludens-1) antibody was from Abcam (UK). Anti-podocin antibody and horseradish peroxidase (HRP)-conjugated goat-anti-rabbit/mouse antibody were from Sigma (USA). Anti-nephrin antibody was from Prosci (USA). Anti-RhoA antibody and anti-tubulin antibody were from Cell Signaling Technology (USA). Anti-ROCK1 antibody and anti-GAPDH antibody were from Proteintech (USA).

### Animal models and specimen collection

All experimental procedures were approved by the Animal Care Center of Third Military Medical University, and complied with the Care and Use of Laboratory Animals published by the U.S. National Institutes of Health. Six-week-old male C57BL/6J mice were obtained from the Laboratory Animal Center of the Third Military Medical University. After 12 h of fasting, 8-week-old mice were intraperitoneally injected with Streptozotocin (160 mg/kg, dissolved in 0.1 mmol/l citrate buffer, pH 4.5, Sigma, USA) to induce Type 1 diabetes mellitus or Streptozotocin (50 mg/kg) to induce Type 2 diabetes mellitus after high food diet. Diabetes was confirmed with random blood glucose levels higher than 16.7 mmol/l 72 h later. Mice injected with citrate buffer served as the vehicle control group. Mice were intravenously injected with Recombinant mouse PEDF protein (8 × 10^−5^ µmol/g/day for 5 days, Sino Biological Inc., China) at the fourth week after diabetes establishment. Mice were kept in individual metabolic cages to collect 24-h urine before anesthesia, and the centrifuged urine was stored to assess the urinary microalbumin and creatinine. Blood samples were obtained to measure the levels of serum PEDF and serum creatinine. Kidneys were removed for hematoxylin–eosin (HE) staining and transmission electron microscopy.

### Cell culture and processing

The conditionally immortalized mouse podocyte cell line was obtained from the Cell Resource Center (Peking Union Medical College, China). Podocytes were cultured as previously described [16], in RPMI-1640 medium containing 10% Fetal bovine serum (HyClone, USA) and 10 U/ml IFNγ (Invitrogen, USA) at 33 °C for proliferation. After switching the cells to a medium without IFNγ at 37 °C, the cells began to differentiate for 10–14 days. Before further processing, differentiated cells were starved in DMEM (HyClone) with 0.1% FBS containing 5.5 mM glucose for 24 h. Then, cells were cultured in normal glucose (NG) medium (5.5 mM glucose + 24.5 mM mannose) or high glucose (HG) medium (30 mM glucose). Then cells were incubated with PEDF (1000 ng/ml) for 24 h, and C3 transferase (10 µg/ml, Sigma) was added at the final 4 h as required.

### Measurements of urinary albumin and creatinine

Urinary albumin was measured by using commercially available Microalbumin Assay Kits (Shanghai Biosun, China), and urinary creatinine was measured using the Jaffe method with the Creatinine Assay Kit (Sigma), following the manufacturer’s protocol.

### ELISA assay

The serum PEDF level was measured using commercially available PEDF ELISA kits (USCN Life Science, China) according to the manufacturer’s protocol.

### Histology and ultrastructure analysis

Mice kidney sections were fixed, dehydrated, and embedded in paraffin. The tissues were cut into 4-µm-thick sections and stained with HE. The volume of the glomeruli and capsular spaces was calculated as previously described [16]. For ultrastructural observation, the renal cortex was cut into fragments and stabilized in glutaraldehyde, followed by dehydration. Ultrathin sections were stained and visualized with a transmission electron microscope (Philips Electron Optics, the Netherlands).

### Podocyte permeability assay

Differentiated podocytes (1 × 10^5^) were plated in the upper transwell chamber, grown to a confluent layer, and serum-starved for 24 h. After PEDF stimulation, FITC-labeled dextran (Sigma) was added to the upper well. Aliquots were collected from the lower layer one hour later, transferred to a 96-well plate, and measured with the plate reader (Thermo Fisher Scientific, USA) at 490/510 nm.

### F-actin cytoskeleton fluorescence staining

Differentiated podocytes were grown on laminin-coated glass cover slips and then fixed in 4% paraformaldehyde, permeabilized with 0.1% Triton X-100, blocked with 1% bovine serum albumin, and stained with Phalloidin (Molecular Probes, USA). Cells were observed using a confocal laser scanning microscope (Leica, Germany).

### Apoptosis assay

Apoptosis of podocytes was assessed with an Annexin V-Cy3 Apoptosis Kit Plus (BioVision, USA) and analyzed by NovoCyte flow cytometry (ACEA Biosciences, USA). Annexin V+/SYTOX− (early stages of apoptosis) is represented in the lower right quadrant, whereas Annexin V+/SYTOX+ (late apoptotic stage or secondary necrotic cells) is shown in the upper right quadrant.

### RhoA pull-down assay

RhoA activity was evaluated with the RhoA Pull-down Activation Assay Biochem Kit (Cytoskeleton, USA) according to the manufacturer’s instruction. Briefly, cells were washed with PBS and lysed in Cell Lysis Buffer containing 1 × Protease Inhibitor Cocktail on ice. Cell lysates were immediately collected into pre-labeled sample tubes, and centrifuged at 10,000×*g*, 4 °C, 1 min. Extraction was performed using 300 µg (0.5 mg/ml) cell lysate, which was incubated with rhotekin Rho-binding domain beads (50 µg) on a rocker, 4 °C, 1 h, and then centrifuged at 5000×*g*, 4 °C, 1 min. The beads were washed with Wash Buffer, centrifuged at 5000×*g*, 4 °C, 3 min, and then boiled for 2 min in 20 µl of 2 × Laemmli sample buffer. The samples were analyzed by Western blot.

### Western blot assay

The WB protein from kidney tissue samples or podocytes was prepared by using extraction and concentration detection kit (Beyotime, China). After being separated by SDS-PAGE, proteins were transferred to PVDF membranes (Millipore, USA), blocked with 5% skimmed milk, and incubated with the corresponding primary and secondary antibodies. Specific protein bands were detected using chemiluminescence (GE Healthcare, UK) and the ChemiDoc XRC^+^ Imaging System (Bio-Rad Laboratories, USA).

### Statistical analysis

All the data are expressed as the mean ± SEM or median and in the interquartile range. Statistically significant differences between groups were assessed using a two-tailed one-way ANOVA followed by Tukey’s post-hoc analysis, or the Kruskal–Wallis *H* test as required. A *P* value < 0.05 was considered statistically significant.

## Results

### Elevated serum PEDF was positively correlated with proteinuria in diabetic mice

**Fig. 1:**
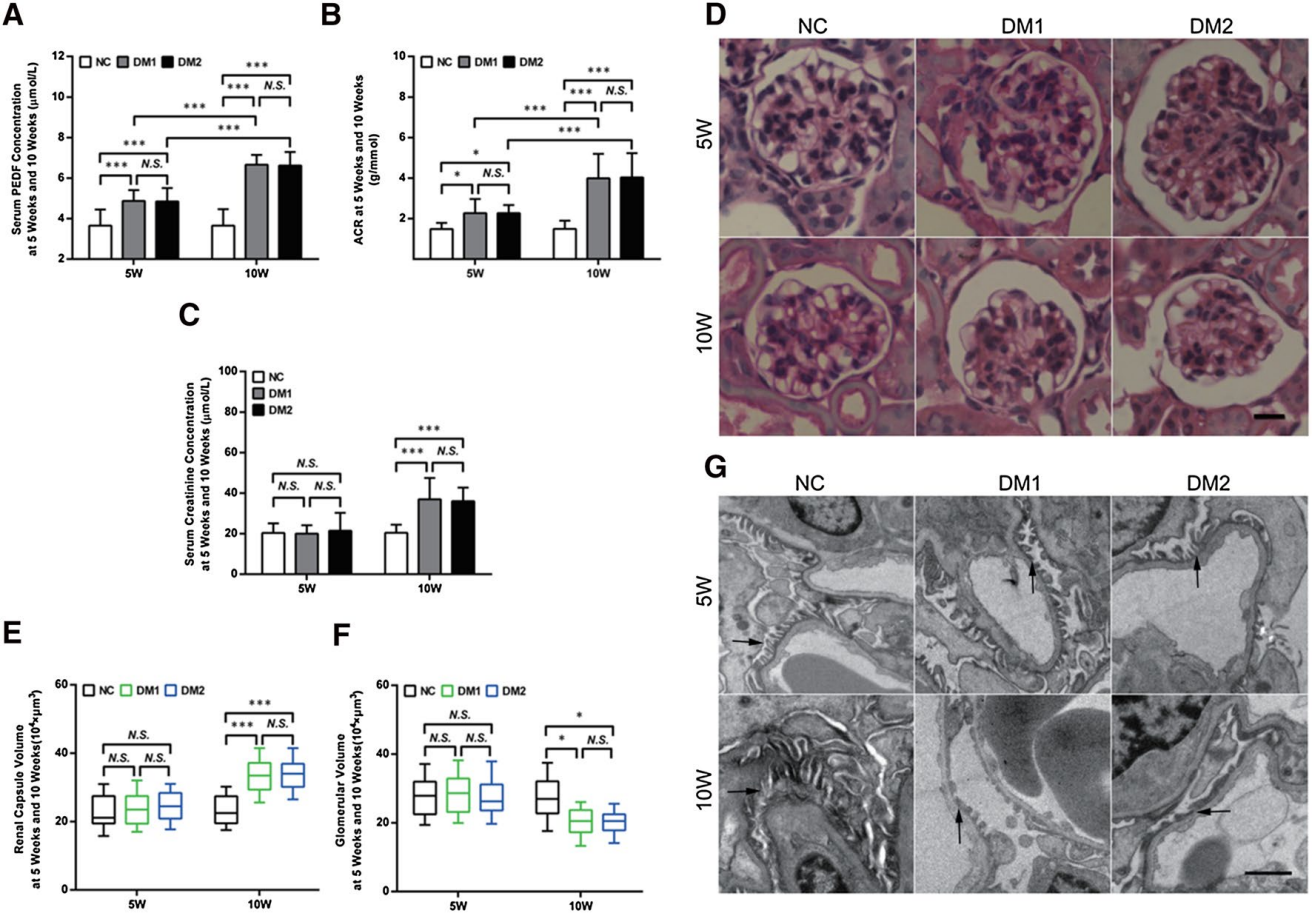
Elevated serum PEDF was positively correlated with proteinuria in diabetic mice. **a** Serum PEDF levels, **b** urinary ACR levels, and **c** serum creatinine levels in different groups. *n* = 15 in NC 5w, DM1 5w, and DM2 5w; *n* = 8 in NC 10w, DM1 10w, and DM2 10w. **d**–**f** Representative microscopy images of HE staining of glomeruli in different groups. Original magnification 400×, scale bar = 25 µm. **g** Representative transmission electron microscopy images of podocyte foot process in different groups (foot processes indicated by black arrows). Original magnification 2000×, scale bar = 1000 nm. The data are shown as the mean ± SEM. ***P < 0.001, *P < 0.05. *N.S*. no significant difference

Figure 1a shows that diabetic mice exhibited significantly elevated levels of serum PEDF at the 5th week after diabetes induction compared with normal mice. The serum PEDF levels of mice at the tenth week after diabetes induction were significantly elevated compared with the mice of 5 weeks, which indicates that serum PEDF levels continued to increase, accompanied by the development of diabetes. No significant difference in serum PEDF levels was observed between mice with Type 1 (DM1) and Type 2 diabetes mellitus (DM2) both at 5 or 10 weeks after diabetes induction.

Correspondingly, the albumin-to-creatinine ratio (ACR) of diabetic mice was significantly increased at the fifth week (Fig. 1b), while the serum creatinine concentration (Fig. 1c) showed no significant difference. At the tenth week, both ACR (Fig. 1b) and serum creatinine concentration (Fig. 1c) were significantly elevated in diabetic mice compared with those of the mice at the 5th week after diabetes induction, indicating a further decline of renal function. No significant difference in ACR was observed between Type 1 and Type 2 diabetic mice at 5 or 10 weeks after diabetes induction (Fig. 1b, c).

An increase in the volume of the capsular space at 10 weeks after diabetes induction could be observed under light microscopy with HE staining, while no significant difference appeared compared with normal mice at 5 weeks (Fig. 1d–f). The ultrastructural changes of the glomeruli were further observed by TEM (Fig. 1g), and the podocytes appeared with FPE at 5 weeks after diabetes induction, and worsened in 10 weeks. Elevated serum PEDF was positively correlated with proteinuria, increased creatinine concentration, and increased FPE, indicating that serum PEDF may play a role in the development of diabetic kidney disease.

### PEDF promoted proteinuria in both normal and diabetic mice

**Fig. 2:**
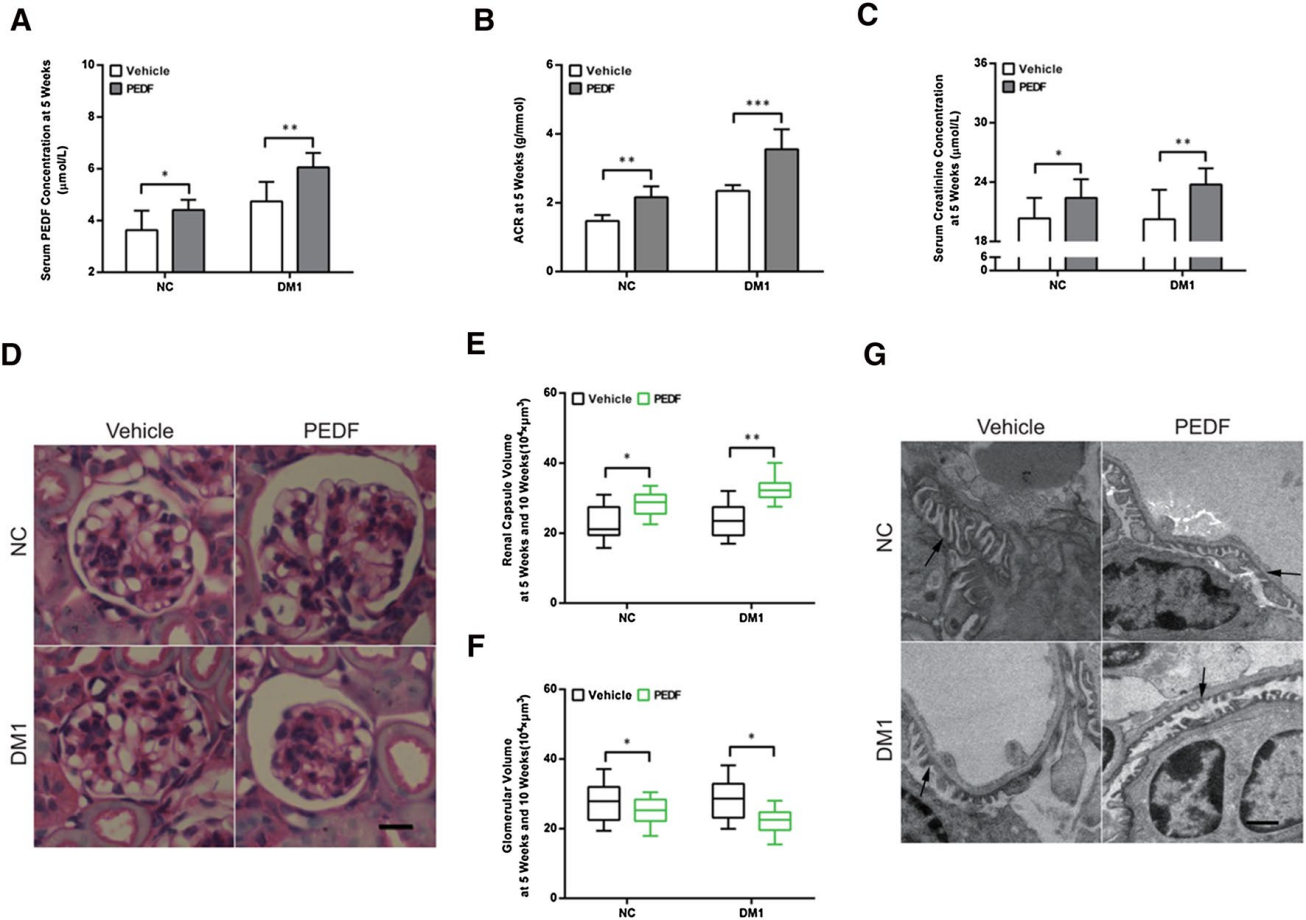
PEDF promoted proteinuria development in both normal and diabetic mice. **a** Serum PEDF levels, **b** urinary ACR levels, and **c** serum creatinine levels in different groups. *n* = 6 in each group. **d**–**f** Representative microscopy images of HE staining of glomeruli in different groups. Original magnification 400×, scale bar = 25 µm. **g** Representative transmission electron microscopy images of podocyte foot process in different groups (foot processes indicated by black arrows). Original magnification 2000×, scale bar = 1000 nm. The data are shown as the mean ± SEM. ***P < 0.001, **P < 0.01, *P < 0.05

To further evaluate the effect of elevated serum PEDF on proteinuria development in diabetic mice, exogenous PEDF was intravenously injected at the 4th week of diabetes induction for five continual days to mimic elevated endogenous serum PEDF. The results showed that serum PEDF levels were significantly increased at the fifth week of diabetes induction after PEDF protein injection compared with non-injected mice in the normal and Type 1 diabetic mouse groups (Fig. 2a).

Correspondingly, ACR and serum creatinine were elevated significantly compared with non-injected mice in the normal and diabetic mouse groups (Fig. 2b, c), and the volume of the capsular space increased while the glomeruli volume clearly decreased, indicating the atrophy of the glomeruli after PEDF treatment (Fig. 2d–f). Additionally, FPE could be observed in normal mice, and appeared to worsen in diabetic mice after serum PEDF treatment (Fig. 2g). Overall, these results demonstrated that increasing levels of serum PEDF contributed to the proteinuria and kidney damage in both normal and Type 1 diabetic mice.

### PEDF led to actin rearrangement and apoptosis of podocytes

**Fig. 3:**
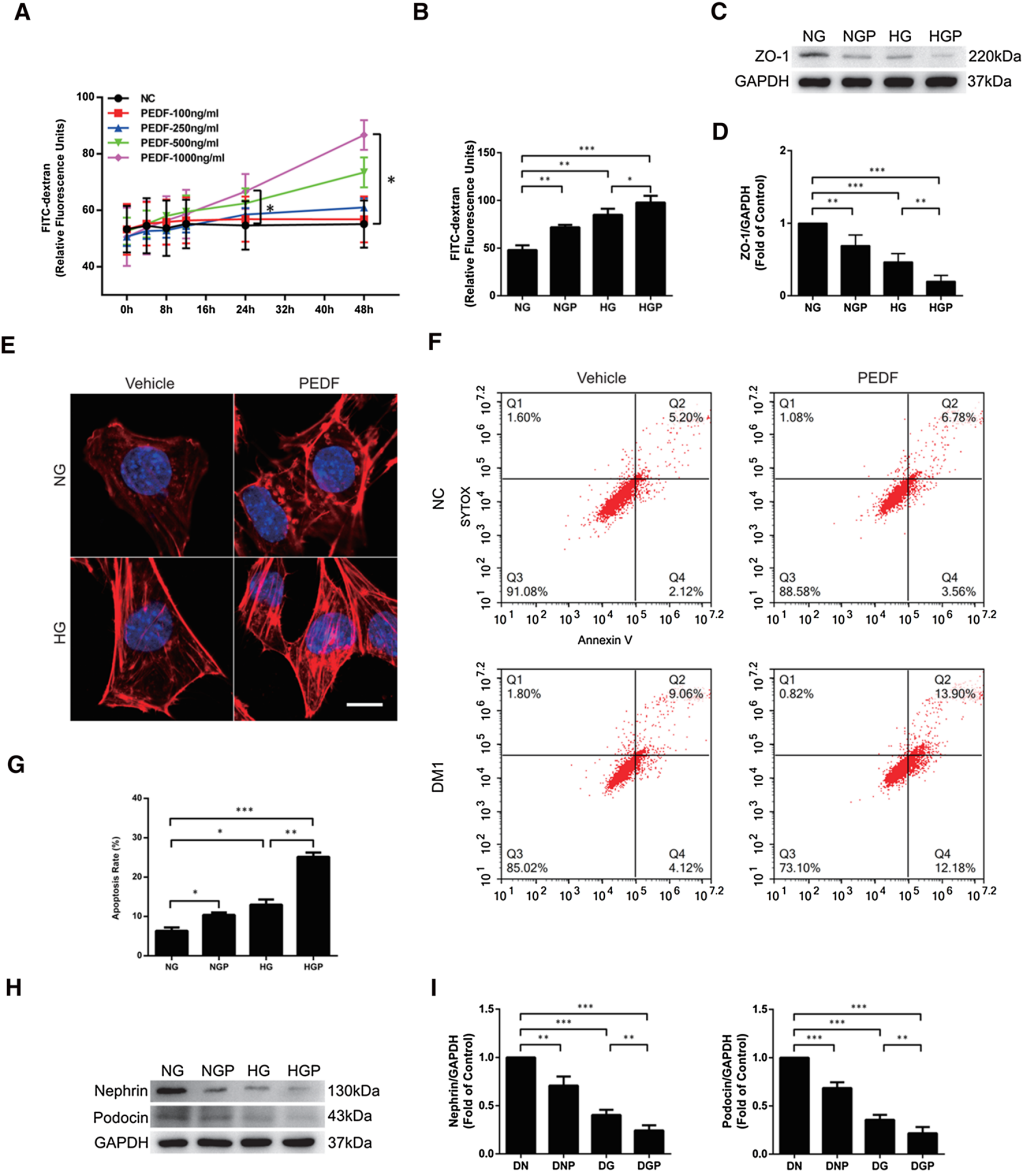
PEDF led to actin rearrangement and apoptosis of podocytes. **a** Paracellular permeability was measured 1 h after the addition of FITC-dextran. Podocytes were treated with PEDF at concentrations of 100, 200, 500, and 1000 ng/ml at different hours. **b** Podocyte paracellular permeability was measured in different groups. **c, d** Western blot was used to detect ZO-1 expression of cultured podocytes in different groups. **e** Podocytes were stained for F-actin (red). Scale bars = 10 µm. **f, g** Annexin V-Cy3/SYTOX double-staining labeled podocytes in each group and flow cytometry was used to determine the podocyte apoptosis rate. Cells in the early stage of apoptosis are Annexin V+/SYTOX−, while cells in late apoptosis or necrotic cells are Annexin V+/SYTOX+. **h, i** Western blot was used to detect nephrin and podocin expression of cultured podocytes in different groups. The data are shown as the mean ± SEM. ***P < 0.001, **P < 0.01, *P < 0.05

It has been reported that podocytes play a key role in proteinuria development [17]. To more directly illuminate whether the effect of PEDF on proteinuria development is exerted through the podocytes, the effect of PEDF on podocytes was detected in vitro. PEDF induced an increase in FITC-dextran leakage in a dose- and time-dependent manner, and the most significant dose was 1000 ng/ml at 24 and 48 h (Fig. 3a).

Furtherly, podocytes exposed to high glucose led to FITC-dextran leakage and ZO-1 expression decline compared with normal glucose, and additional stimulation with PEDF aggravated the changes (Fig. 3b–d).

Considering that extensive FPE was observed in the podocytes after PEDF treatment (Fig. 2g), and the FPE and paracellular permeability increase of podocytes were closely associated with F-actin, we observed the changes in actin distribution following PEDF stimulation. The results (Fig. 3e) show significantly denser F-actin filaments after high-glucose or PEDF stimulation compared with the long thin F-actin fibers in the normal group. In cells co-stimulated with high glucose and PEDF, even thicker and denser, wider F-actin appeared.

Moreover, PEDF promoted apoptosis under normal glucose conditions and exacerbated apoptosis when subject to high-glucose stimulation (Fig. 3f, g), which was in line with WB analysis of nephrin and podocin (specific protein markers of podocytes, Fig. 3h, i). These findings identify PEDF as a modulator of paracellular permeability of podocytes through regulation of F-actin rearrangement and apoptosis.

### RhoA/ROCK1 signaling pathway mediated actin rearrangement in PEDF-treated podocytes

**Fig. 4:**
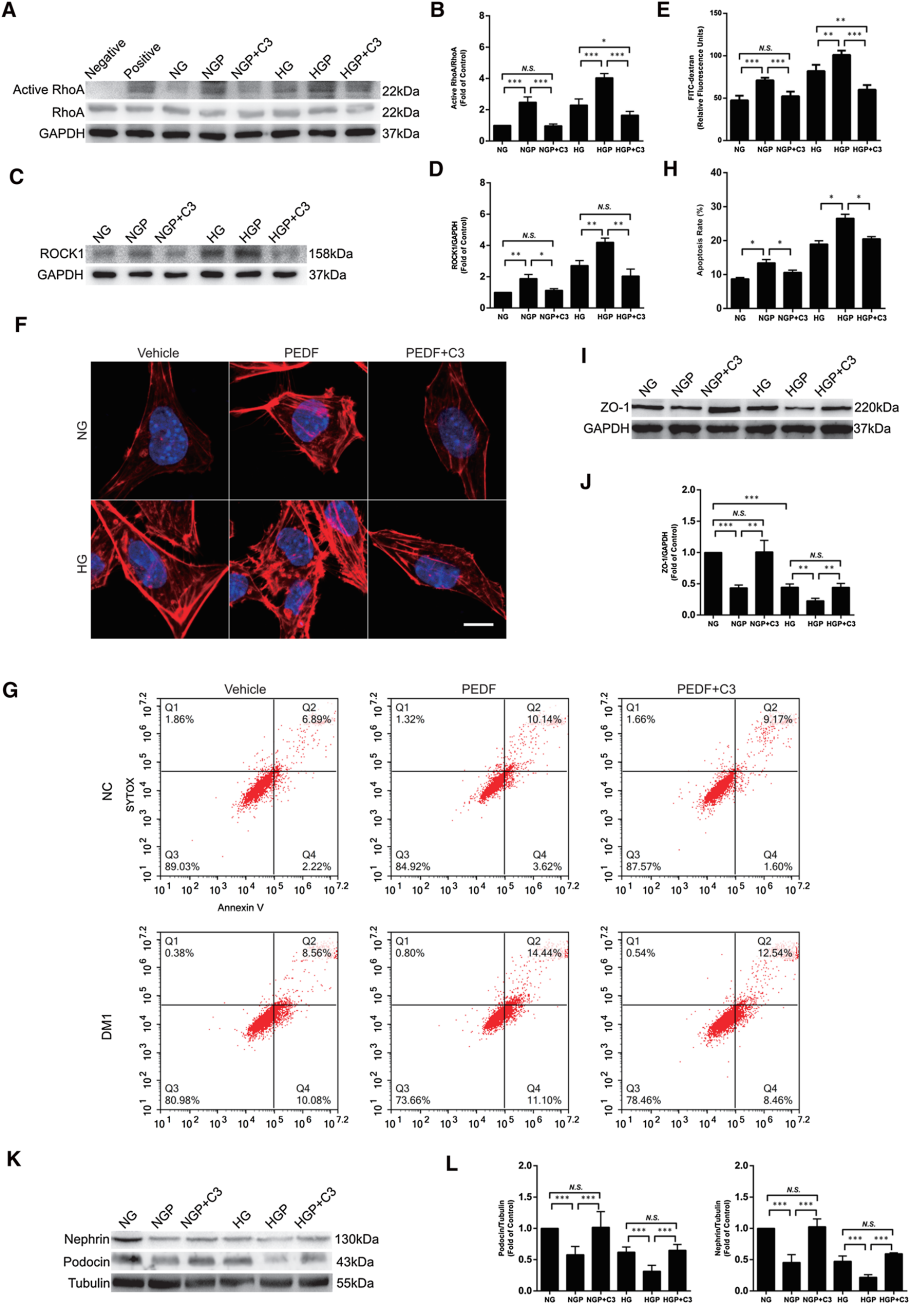
The RhoA/ROCK signaling pathway mediated actin rearrangement in PEDF-treated podocytes. **a, b** RhoA pull-down assay and western blot was used to detect RhoA activity and total RhoA expression of cultured podocytes in different groups. **c, d** Western blot was used to detect ROCK1 expression of cultured podocytes in different groups. **e** Paracellular permeability was measured 1 h after the addition of FITC-dextran in each group. **f** Podocytes were stained for F-actin (red). Scale bars = 10 µm. **g, h** Flow cytometry was used to determine the apoptosis of Annexin V/SYTOX double-staining labeled podocytes in each group. **i**–**l** Western blot was used to measure ZO-1 (**i, j)**, nephrin, and podocin (**k, l**) expression of cultured podocytes in different groups. The data are shown as the mean ± SD. ***P < 0.001, **P < 0.01, *P < 0.05, *N.S*. no significant difference

RhoA has been recognized as the most important regulator in stress fiber formation [5]. We therefore analyzed whether RhoA was involved in F-actin formation in PEDF-treated podocytes. The results showed that RhoA activity significantly increased in podocytes pretreated with high glucose or PEDF or co-stimulated podocytes, and significantly decreased after C3 transferase (RhoA inhibitor) treatment (Fig. 4a, b). The expression of RhoA remained the same (Fig. 4a). ROCK1, the downstream effector of Rho GTPase activation, was also activated and obviously blocked by C3 transferase (Fig. 4c, d).

Correspondingly, C3 transferase alleviated the effect of PEDF and (or) high glucose on podocytes, indicating an increase in paracellular permeability (Fig. 4e), stress fiber formation (Fig. 4f), and cell apoptosis (Fig. 4g, h). C3 transferase also restored the expression of ZO-1, nephrin, and podocin protein (Fig. 4i–l). Taken together, these results suggest that RhoA/ROCK1 might be the key pathway mediating the actin rearrangement and apoptosis in PEDF-treated podocytes.

## Discussion

In the current study, we found that elevated serum PEDF promoted FPE, and thus induced and worsened proteinuria in normal and diabetic mice in vivo; elevated PEDF was also responsible for rearranging F-actin, increasing paracellular permeability, elevating apoptosis, and reducing the expression of ZO-1, nephrin, and podocin in podocytes in vitro. Moreover, our results showed that the RhoA/ROCK1 signaling pathway, being triggered after PEDF stimulation, might be responsible for PEDF-induced effects and was blocked by RhoA inhibitor.

Albuminuria/proteinuria is a hallmark of damage to the GFB. Podocytes, the final layer of GFB, interdigitate with adjacent cellular foot processes and are connected by slit diaphragm. Either shape or number change of podocytes can lead to albuminuria [18]. It is generally accepted that FPE is generally accepted as a typical morphological alteration of podocytes related to cytoskeletal rearrangement and is closely associated with glomerular filtration disorder, together with podocyte apoptosis [17].

The serum PEDF level is found to be elevated in patients with metabolic syndrome, diabetes mellitus, atherosclerosis, or polycystic ovary syndrome [9, 19–21]. The elevated PEDF in patients with diabetes was found to be associated with poor vascular health [10]. In the current study, we found serum PEDF levels were significantly elevated in diabetic mice, in accordance with previous studies [7, 9, 10]. We also found prolonged PEDF treatment (8 × 10^−5^ µmol/g/day for 5 days) as previously described [22] aggravate podocyte FPE, thus inducing proteinuria in normal mice, or exacerbated proteinuria in diabetic mice. These results contradict previous studies that reported that PEDF peptide ameliorated proteinuria and played a protective role in diabetic renal disease [23–25].

The reason for this contradiction may be due to the difference in the PEDF dosage applied in previously conducted studies. Apte et al. demonstrated PEDF at low concentration inhibited the neovasculature, whereas high doses stimulated angiogenesis possibly due to PEDF activating receptors differing in their ligand affinity and thus leading to completely different results [22]. Our study showed that the concentration of serum PEDF was 1.3 times higher in diabetic mice at the fifth week of diabetes induction, and climbed to 1.8 times higher at the tenth week (Fig. 1a), which was consistent with a previous study [13]. The dosage of PEDF protein applied in the current study elevated the serum PEDF to levels that were similar to those in diabetic mice, which was much higher than that applied in the other studies (1.7 × 10^−5^ µmol/g/day for 6 weeks) [24]. The results demonstrated that a dosage of PEDF similar to that of the diabetic mice promoted proteinuria development in both normal and diabetic mice. Except for the different dosage, PEDF may exert different biological actions by binding to different cell-surface receptors through various functional domains [8], and thus, we speculate that the full length PEDF we applied and the PEDF peptide applied in the previous study may lead to different results. Meanwhile, the dosage of PEDF we chose (1000 ng/ml) to stimulate the cultured podocytes was much higher than that applied in other studies (1–10 nM) [26]. This is similar to the animal experiment results of PEDF that low-dosage PEDF showed protective effects, whereas high dose disrupted.

FPE of podocytes is highly dependent on actin-based cytoskeletal rearrangement. The Rho family of small GTPases is tightly associated with the regulation of actin cytoskeleton, cell junction, and cell migration [27]. In the past decade, the small GTPase RhoA has been implicated as a potent regulator of proteinuria [16, 28]. Activation of RhoA results in FPE, decreasing actin-associated protein, podocyte apoptosis, focal segmental glomerulosclerosis, and fibronectin induction. In this study, we tested whether the RhoA/ROCK pathways were involved in the PEDF-stimulated hyperpermeability and apoptosis of podocytes. We found that the activity of RhoA was increased after high glucose or PEDF treatment in podocytes, the same as its downstream kinase ROCK1, and the RhoA inhibitor C3 transferase could block the PEDF-induced effects in podocytes, including actin rearrangement, paracellular permeability increase, and cell apoptosis. These findings suggest that the RhoA/ROCK1 pathway activated by PEDF may play a critical role in PEDF-induced proteinuria.

In summary, the present study demonstrated that elevated serum PEDF aggravated the development of proteinuria and renal dysfunction in diabetic mice through promotion of actin arrangement and apoptosis of podocytes via activating the RhoA/ROCK1 signaling pathway. The RhoA inhibitor blocked the PEDF-induced effects in podocytes, which suggests that inhibition or antagonism of serum PEDF may provide a new potential therapeutic strategy for proteinuria in DKD patients.
